# Factors Affecting Quality of Life among Older Adults with Hypertension in Urban and Rural Areas in Thailand: A Cross-Sectional Study

**DOI:** 10.1177/00914150211050880

**Published:** 2021-12-21

**Authors:** Chonticha Chantakeeree, Marjorita Sormunen, Matti Estola, Pornchai Jullamate, Hannele Turunen

**Affiliations:** 1 Department of Nursing Science, Faculty of Health Sciences, 101232 University of Eastern Finland, Box 1627, 70211, Kuopio, Finland; 2 Gerontological Nursing Division, Faculty of Nursing, 37688 Burapha University, 169 Long-Hard Bangsaen Road, Tambon Saensook, Amphur Muang, Chonburi, 20131, Thailand; 3 Institute of Public Health and Clinical Nutrition, Department of Medicine, Faculty of Health Sciences, 205537 University of Eastern Finland, Box 1627, 70211, Kuopio, Finland; 4 Faculty of Social Sciences (Joensuu Campus), 122208 University of Eastern Finland, Box 111, 80101, Joensuu, Finland; 5 60650 Kuopio University Hospital, Box 100, 70029 KYS

**Keywords:** quality of life, older adults, hypertension, urban, rural

## Abstract

This study explored factors affecting quality of life in older adults with hypertension by comparing those living in urban and rural areas. A cross-sectional study was conducted on 420 older adults living in urban and rural areas in Thailand. Data were collected using the WHOQOL-OLD and Health-Promoting Lifestyle Profile-II tools, which measured quality of life and health-promoting behaviors among the participants. Older adults in urban areas had higher quality of life scores than those in rural locations. Health-promoting behaviors significantly predicted higher quality of life for all residents. A high perceived health status predicted increase of quality of life in urban residents, whereas the presence of comorbidity effects decreased quality of life. A longer hypertension duration predicted higher quality of life in rural residents. These findings suggest that healthy behaviors and self-management interventions are critical to improve quality of life in older Thai adults with hypertension.

## Introduction

Hypertension is a serious disease and a major global health problem (Benjamin et al., 2019). The prevalence of hypertension dramatically increases in association with increasing age (van Bussel et al., 2020). The World Health Organisation (WHO) has estimated the number of people aged 60 years and over will increase from 1 billion to 1.4 billion between the years 2019 and 2050 (WHO, 2020). Approximately 60% to 70% of older adults currently suffer from hypertension, which is the main cause of stroke and ischemic heart disease, leading to premature deaths worldwide (WHO, 2019). A nationwide survey in Thailand indicated that over 60% of older people are living with hypertension and poorly controlled blood pressure (Meelab et al., 2019; Sakboonyarat et al., 2019; Woodham et al., 2018). Therefore, given the prevalence among older adults, hypertension is likely to have a significant influence on quality of life (QoL) in older adults (Benetos et al., 2019).

The QoL of older adults with hypertension has been found to be lower than in healthy individuals, and a reduction in QoL has been identified as due to the symptoms of the disease itself (Gu et al., 2019; Heidari et al., 2019). According to the WHO (2020), QoL is an important indicator for overall health, meaning the state of perceived complete physical and mental health and social well-being, not merely the absence of disease and infirmity. Hypertension may lead to mental decline from various pathophysiological and psychological changes that disturb older adults’ everyday life activities. (Haraldstad et al., 2019; Sekeon et al., 2017).

Several studies have been conducted to evaluate the relationship between associating factors and QoL in older adults. Sociodemographic factors display a disparity and may affect QoL among older adults with hypertension (dos Santos Tavares et al., 2015). Increasing age, living alone, and lower education are more likely to be associated with lower QoL (Bhandari et al., 2016). In addition, an increase in the number of chronic conditions is associated with decreased QoL in older adults (Chen et al., 2020), as higher multimorbidity may affect the severity of the disease leading to less ability to undertake everyday activities, greater social isolation, and lower well-being (Dev et al., 2020). Furthermore, financial limitations for transportation and other expenses may decrease access to healthcare services and restrict lifestyle choices, which in turn may affect poor health conditions and reduce life satisfaction in older adults (Ong-Artborirak & Seangpraw, 2019). The duration of hypertension, in particular, is one of the factors related to QoL; if an older adult has suffered high levels of hypertension for a long time, it affects physical conditions and may lead to poor adherence to treatment resulting in negative health outcomes and poor QoL in old age (Uchmanowicz et al., 2018). However, perceived health status regarding age-related disease may influence older adults’ self-care efforts to retain healthy habits and medication adherence, thus enhancing their QoL (Gu et al., 2019).

The major strategies for promoting and supporting QoL cover the multidimensional aspects of older persons’ lives in terms of healthy ageing (Manasatchakun et al., 2016). Hence, health-promoting behaviors (HPBs) are strongly recommended for hypertensive older adults for controlling hypertension and lead to improvement of QoL (Manasatchakun et al., 2016; Oliveros et al., 2020; Williams et al., 2018). Pender et al. (2015) described that HPBs are directed towards increasing prosperity and achieving positive health outcomes, in particular when integrated into a healthy lifestyle, such as health responsibility, physical activity, nutrition, spiritual growth, interpersonal relations, and stress management. Considering geographical characteristics, one's living environment is one of the factors that influence lifestyle and therefore is important for assessing individual QoL (Hancock & Wells, 2019).

Residential areas are associated with hypertension prevalence, lifestyle, and QoL among older adults (Hancock & Wells, 2019; Laohasiriwong et al., 2018). Prior studies have assessed the relationship between QoL and residential settings among older adults, but findings have not been consistent (Hsu et al., 2019; Usha & Lalitha, 2016). Zhang et al. (2019) found that urban older Chinese adults with chronic disease were more likely to seek healthcare and improve their QoL when compared with older adults from rural locations. Similarly, Polish and South-African studies demonstrated a higher QoL in urban older adults than their rural counterparts (Kaczmarek et al., 2017; Peltzer et al., 2019). Conversely, rural communities have several disadvantages for older people, including socioeconomic limitations and a lack of public healthcare services due to fewer healthcare professionals compared with an urban setting, all of which affect the QoL of rural older adults (Seangpraw et al., 2019; Yodmai et al., 2018). However, studies in Thailand and Iran revealed that the QoL of older Thai and Iranians did not differ when comparing those living in urban and rural areas (Heidari et al., 2019; Yiengprugsawan et al., 2012).

Understanding the factors that affect one's lifestyle and quality of life in different residential locations may help healthcare personnel in creating and implementing individualized interventions to improve the QoL of older adults according to cultural contexts. Although previous studies have compared QoL in older adults living in urban and rural areas, the factors that affect QoL in different residential settings remain unclear (Woodham et al., 2018). This study aimed to evaluate and compare factors affecting QoL among older adults with hypertension living in different residential areas. We hypothesized that: (a) the QoL of older adults with hypertension living in urban and rural areas would differ, and (b) the factors affecting QoL, such as sociodemographic characteristics and HPBs would vary based on residential environment.

## Methods

### Participants

A cross-sectional, comparative study was carried out in one province in the eastern region of Thailand. The study participants were older Thai adults who registered as a member of the elderly clubs. The inclusion criteria were adults aged 60 years and over with hypertension taking anti-hypertensive medication without complications. Participants’ age was classified and presented into three groups: 60–69 years, 70–79 years, and 80 years and above (Foundation of Thai Gerontology Research and Development Institute [TGRI], 2019).

The cognitive impairment was assessed by the Mini-Mental State Examination–Thai 2002. Participants who had a risk of cognitive impairment and had any symptoms of a hypertensive crisis (e.g., severe headache, dizziness, blurred vision, nausea, and vomiting) were excluded from the study. A multistage, random sampling method was used for study sample selection (Polit & Beck, 2017). In the first stage, based on community characteristics of a province, all 11 districts were divided into two categories of urban and rural areas. Based on the National Statistical Office Report (2020), urban refers to inside municipalities which are inhabited by at least 200 persons per square kilometer, whereas rural is indicative of being outside of the municipalities with a population density lower than 200 persons per square kilometer. Following this definition, a total of 52 subdistricts within the urban and rural areas were identified and included in the second stage. A random selection of 12 subdistricts where elderly clubs were located was taken, which resulted in a sample of eight elderly clubs in urban subdistricts and four in rural subdistricts. At the last stage, registered members of these clubs were randomly selected and invited to participate in the study. The proportion of the samples from urban and rural areas was 70:30 based on a representative population size in both locations. An appropriate sample size was determined using a power of 80%, confidence interval of 95%, and acceptable error of 5%. A total of 420 participants with complete questionnaires were accepted. There were 297 urban older adults with mean age was 70.20 (SD  =  6.55, range  =  60–89), and 123 rural older adults, mean age was 70.15 (SD  =  7.13, range  =  60–86).

The sociodemographic characteristics of the participants (N = 420) are presented in Table 1. The mean age was 70.18 years (SD  =  6.71, range  =  60–89) and 81.9% were female. The duration of hypertension averaged 7.03 years (SD  =  7.00, range  =  1–50), and almost half presented with comorbidity of at least one disease (44.0%). Over half of the participants were without a partner (52.6%), and the majority lived with another person (86.4%). Two-thirds (66.2%) of participants had a primary school education, almost three-quarters (73.8%) were not working or were retired, and 41.0% had an income of less than 32.8 US$ per month. Almost equal percentage rated their perceived health status as fair (47.6%) and good (44.5%). When considering HPBs, the mean score among all participants was 3.04 (SD  =  0.39, range  =  1.98–3.99).

**Table 1.** Comparison of Quality of Life and Sociodemographic Characteristics Between Urban and Rural Areas.

Variables			Quality of life					
	Total		Urban (n = 297)			Rural (n = 123)		
	n	%	Mean (*SD*)	t/F	*p*-value	Mean (*SD*)	t/F	*p*-value
Age (Years) Mean (SD), range	70.18 (6.71), 60-89		70.20 (6.55), 60-89			70.15 (7.13), 60-86		
60–69	206	49.0	3.54 (0.39)	0.934	.394	3.54 (0.33)	.207	.813
70–79	170	40.5	3.61 (0.41)			3.49 (0.41)		
80 and over	44	10.5	3.61 (0.37)			3.53 (0.39)		
Hypertension duration (Years) Mean (SD), range	7.03 (7.00), 1-50		6.87 (6.90), 1-43			7.46 (7.29), 1-50		
< 5	185	44.0	3.58 (0.39)	0.164	.849	3.46 (0.35)	1.869	.159
5–10	180	42.9	3.56 (0.42)			3.55 (0.37)		
> 10	55	13.1	3.61 (0.34)			3.63 (0.37)		
Gender								
Female	344	81.9	3.60 (0.39)	1.86	.06	3.51 (0.35)	− 0.56	.58
Male	76	18.1	3.49 (0.42)		3.57	(0.44)		
Living arrangement								
With family/someone	363	86.4	3.59 (0.40)	−1.35	.18	3.53 (0.37)	−0.88	.38
Alone	57	13.6	3.50 (0.35)			3.44 (0.37)		
Education								
Not educated	28	6.7	3.62 (0.42)	1.37	.26	3.56 (0.59)	0.37	.69
Primary school	278	66.2	3.55 (0.39)			3.53 (0.36)		
Higher than primary school	114	27.1	3.62 (0.40)			3.47 (0.28)		
Working status								
Not working & retired	310	73.8	3.60 (0.39)	1.60	.11	3.52 (0.39)	0.10	.92
Currently working	110	26.2	3.51 (0.40)			3.52 (0.33)		
Marital status								
Married	199	47.4	3.58 (0.42)	0.22	.83	3.58 (0.42)	0.22	.83
Single/widowed/divorced /separated	221	52.6	3.57 (0.37)			3.57 (0.37)		
Monthly income								
< 1,000 baht (<33.3 US$)	172	41.0	3.55 (0.40)	0.91	.40	3.54 (0.40)	0.91	.40
1,000 to 5,000 baht (33.3-166.7 US$)	132	31.4	3.62 (0.44)			3.62 (0.44)		
>5,000 baht (>166.7 US$)	116	27.6	3.58 (0.40)			3.58 (0.40)		
Presence of comorbidity								
No presence	124	29.5	3.50 (0.42)	1.65	.19	3.49 (0.31)	2.73	.07
One disease	189	44.0	3.59 (0.44)			3.62 (0.39)		
≥ Two diseases	107	25.4	3.58 (0.40)			3.52 (0.37)		
Perceived health status								
Good	187	44.5	3.70 (0.36)	15.16	.001**	3.44 (0.32)	3.22	.04*
Fair	200	47.6	3.49 (0.38)			3.56 (0.37)		
Poor	33	7.9	3.35 (0.46)			3.71 (0.46)		
Health-promoting behaviors (HPBs), Mean (SD), range	3.04 (0.39),	1.98-3.99	3.07 (0.41), 1.98-3.94			2.98 (0.34), 2.15-3.77		

*Note.* t/F indicates value obtained by either independent samples t-test (t) or one-way ANOVA (F). * *p* < .05, ** *p* < .01.

### Procedures

This study was approved by the institutional ethical review board of the University of Eastern Finland (Statement 16/2018) prior to data collection. All sub-district health-promotion hospitals responsible for the elderly clubs also granted approval. The researcher provided information regarding the purpose of the study to the participants, and written informed consent was obtained from all the participants. Data were collected from October 2018 to March 2019. The researcher collaborated with the contact nurses in the sub-district health-promoting hospitals who helped to coordinate the identification of potential participants for the study. In the data collection at the elderly clubs, the researcher instructed the participants regarding the questionnaires and read aloud each item of the questionnaires and asked the participants to rate each item by themselves. Approximately 45 min were required to complete the questionnaire.

### Measures

#### Sociodemographic Characteristics

The sociodemographic characteristics of older adults included age, gender, living arrangement, educational level, working status, marital status, income, presence of comorbidities, and perceived health status.

#### Health-Promoting Behaviors (HPBs)

HPBs were measured using the 52-item health-promoting lifestyle profile-II (HPLP-II) developed by Walker et al. (1987). It has been translated into Thai and used in previous Thai studies (Noosorn & Saengngern, 2013; Ong-Artborirak & Seangpraw, 2019; Sriyuktasuth, 2002). The HPLP-II contains the following six subscales (Cronbach alpha, number of items): health responsibility (α  =  .87, 9 items) focuses on the importance of improving one's health and the health of others (e.g., “Discuss my health concerns with health professionals”); physical activity (α  =  .86, 8 items) refers to adhering to regular exercise patterns (e.g., “Follow a planned exercise program”); nutrition (α  =  .75, 9 items) assesses proper meal patterns and daily food choices (e.g., “Choose a diet low in fat, saturate fat, and cholesterol”); spiritual growth (α  =  .89, 9 items) measures ability to develop oneself and attain one's fullest potential (e.g., “Believe that my life has purpose”); interpersonal relations (α  =  .88, 9 items) are defined as social support, intimacy with others, and participation in community activities (e.g., “Spend time with close friends”); and stress management (α  =  .86, 8 items) contains mechanisms used to cope with stress (e.g., “Get enough sleep”). The participants are asked to evaluate their routine engagement in their healthy lifestyle practices by responding how often they partake in HPBs using a four-point Likert scale (ranging from 1  =  never to 4 = routinely). Total possible scores ranged from 52 to 208, with higher scores indicating a higher level of HPBs. Cronbach's alpha coefficient for the entire scale was 0.96.

#### Quality of Life (QoL)

QoL was measured using the WHOQOL-OLD (World Health Organization Quality of Life) tool developed by Power et al. (2005). The English tool was translated into Thai by an expert who was a native Thai speaker and fluent in English. A back-translation from Thai to English was performed at a languages institute and validated by five healthcare professional experts in geriatrics. This tool is a multidimensional measure of quality of life among older adults and consists of 24 items in six domains, with 4 items in each domain: sensory abilities assesses (α  =  .79) sensory functioning and the effect of loss of sensory abilities on QoL (e.g., “To what extent do impairments to your senses (e.g., hearing, vision, taste, smell, touch) affect your daily life”); autonomy (α  =  .90) refers to ability living in advance age independently and being able to make their own decisions (e.g., “To what extent do you feel in control of your future); past, present, and future activities (α  =  .83) explain satisfaction about achievements in life and expectation of future things (e.g., “How happy are you with the things you are able to look forward to”); social participation (α  =  .91) defines as participation in community activities (e.g., “How satisfied are you with your level of activity”); death and dying (α  =  .73) domain relates to concerns, worries, and fears about death and dying (e.g., “How scared are you of dying”); and intimacy (α  =  .84) evaluated ability to have personal and intimate relationships (e.g., “To what extent do you experience love in your life”). Each item was scored on a five-point Likert scale ranging from 1 (not at all) to 5 (an extreme amount). Possible scores ranged from 24 to 120 points, with higher scores indicating better QoL. The alpha coefficient for the whole scale was 0.95.

### Statistical Analysis

Data analyses were performed using IBM SPSS Windows Version 27.0 (Armonk, NY, USA: IBM Corp.). Descriptive statistics were used to analyse the sociodemographic characteristics, HPBs, and QoL. Independent samples t-test and one-way ANOVA tests were used to compare the variables related to QoL based on residential area. Multiple regression was applied to identify factors affecting QoL under consideration by residential areas.

## Results

### Descriptive Statistics and Comparison

Table 1 displays descriptive statistics and a comparison of the variables of interest in this study. An independent samples t-test and one-way ANOVA tests were used to compare the means of QoL in urban versus rural areas among older adults with hypertension related to sociodemographic characteristics and HPBs. QoL based on the perceived health status level of older adults in urban versus rural areas showed a statistically significant difference (*p* < .001 and *p* < .05, respectively). However, other sociodemographic characteristics had no statistically significant difference with QoL.

A comparison of quality of life and its domains among urban and rural older adults is shown in Table 2. The overall QoL mean score was 3.56 (SD  =  0.39, range  =  2.58–4.38), with the subscale of social participation having the highest mean score of 4.27 (SD  =  0.56, range  =  2.25–5.00), followed by autonomy; past, present, and future activities; intimacy; and sensory abilities. Older adults’ perceptions about death and dying had the lowest mean score of 2.08 (SD  =  1.01, range  =  0.00–5.00). An independent samples t-test was conducted to compare means of QoL and subscales based on residential area. The results indicated that older adults living in urban areas (mean  =  3.61, SD  =  0.33, range  =  2.58–4.33) had a statistically significantly higher QoL score compared to older adults in rural areas (mean  =  3.54, SD  =  0.33, range  =  2.58–4.38, *p* < .05). The effect size for this analysis (d  =  0.21) was found to exceed Cohen's convention for a small effect (Cohen, 1988). The domain of death and dying (*p* < .05) was also found to be significantly higher among urban participants than for older adults in rural areas.

**Table 2.** Quality of Life and Subscales of Older Adults Divided According to Residential Areas.

Variables	Total		Urban		Rural		P-value
	(N = 420)		(n = 297)		(n = 123)		
	Mean (*SD*)	Range	Mean (*SD*)	Range	Mean (*SD*)	Range	
Overall quality of life (QoL)	3.56 (0.39)	2.58–4.38	3.61 (0.33)	2.58–4.33	3.54 (0.33)	2.58–4.38	.05*
Sensory abilities	2.85 (0.77)	1.50–5.00	2.84 (0.79)	1.50–4.75	2.79 (0.64)	1.75–5.00	.49
Autonomy	4.19 (0.74)	2.00–5.00	4.25 (0.68)	2.00–5.00	4.20 (0.68)	2.25–5.00	.50
Past, present, and future activities	4.17 (0.57)	2.00–5.00	4.22 (0.49)	2.50–5.00	4.18 (0.52)	2.00–5.00	.48
Social participation	4.27 (0.56)	2.25–5.00	4.32 (0.52)	2.50–5.00	4.31 (0.48)	2.25–5.00	.82
Death and dying	2.08 (1.01)	0.00–5.00	2.14 (1.06)	0.00–5.00	1.95 (0.76)	1.00–4.25	.04*
Intimacy	3.79 (0.78)	1.50–5.00	3.86 (0.75)	1.50–5.00	3.79 (0.71)	1.50–5.00	.35

*Note. p*-value is obtained by independent samples t-test. * *p* < .05.

### Multivariate Regression Analyses Results

Multiple regression analysis is used to test the predictive factors of QoL among older adults in urban and rural areas and is presented in Table 3. The findings supported the hypotheses that the factors affecting QoL varied based on residential areas. In urban areas, the presence of comorbidity, perceived health status, and HPBs all had a significant effect on QoL, together explaining 28.4% of the variance in QoL (F  =  17.871, *p* < .001). The presence of comorbidity decreased the QoL score by 0.132 points as compared to participants with no comorbidity. Those with a high perceived health status (fair and good levels) had a significantly higher QoL score value (0.155 points, *p*  =  .030) as compared to participants with a low perceived health status (poor level). Within rural areas, the duration of hypertension and HPBs had a significant effect on QoL, together explaining 21.7% of the variance in QoL (F  =  5.123, *p* < .001).

**Table 3.** Multiple Regression for Factors Predicting Quality of Life among Urban and Rural Older Adults (N = 420).

Factors	Urban					Rural				
	Model 1					Model 2				
	B	SE	*β*	*t*	*p*	B	SE	*β*	*t*	*p*
(Constant)	1.947	.165		11.816	.000***	2.175	.311		7.000	.000***	
Duration of hypertension	−.001	.004	-.012	-.231	.817	.010	.006	.151	1.722	.048*
Income											
< 1,000 (Ref)											
1,000 to 5,000	.066	.043	.088	1.523	.129	−.076	.071	-.102	−1.065	.289
> 5,000	.005	.042	.007	.129	.897	.004	.080	-.005	-.055	.956
Presence of comorbidity	−.132	.042	.167	3.153	.002**	−.052	.062	.074	.835	.406
Perceived health status	.155	.071	.112	2.176	.030*	.111	.109	-.090	−1.017	.311
Health-promoting behaviors	.447	.046	.497	9.639	.000***	.462	.100	.411	4.606	.000***
	R = .533, R² = .284, adjusted R² = .268,					R = .466, R²= .217, adjusted R² = .175,				
	F = 17.871 (6, 270), *p* = .000					F = 5.123 (6, 111), *p* = .000				

*Note.* * *p* < .05, ** *p* < .01, *** *p* < .001.

## Discussion

The present study highlights several important findings related to QoL of older adults living in different residential areas in Thailand. First, older adults living in urban areas reported higher QoL than those living in rural areas. Second, HPBs was a statistically significant predictor of QoL in both areas. Third, the presence of comorbidity and perceived health status were statistically significant predictors of QoL for urban residents, whereas duration of hypertension was a statistically significant predictor of QoL for rural residents. These findings support the study hypotheses that QoL and factors affecting QoL differ between older adults living in urban and rural areas.

In this study, urban residents with hypertension reported a higher QoL than rural residents. The significant difference in QoL between older Thai residents in urban and rural settings is consistent with prior studies in other countries. You et al. (2019) indicated that Chinese older adults living in urban areas had better QoL than those in rural areas. Urban–rural disparities were reported, with urban residents typically better in economic status and support from family, leading to a positive influence QoL of elderly people. The traditional cultural expectation in China is that children are expected to take care of their older parents, which is usually respected as a core family role; this cultural expectation is also present in Thailand. A study by Moss et al. (2021) reported that American older adults with cancer had a higher QoL in urban areas versus rural areas, which may relate to health practices and social support that vary by environmental context. Also, previous studies have suggested that in urban areas the QoL of older adults is connected to economic well-being and perceived increasing opportunities for receiving health service, social security, and participation (Rondón García & Ramírez Navarrro, 2018; Stephens et al., 2019; Supromin & Choonhakhlai, 2017; Tiraphat et al., 2017). Our findings concur with previous studies indicating that older residents perceiving more social interaction within their own neighbourhood can help them to release stress, thereby improving their QoL (Gobbens & van Assen, 2018; Neri et al., 2018; Shou et al., 2018). Further, a majority of older adults in our study were living with others, such as their children or relatives. This reflects the Thai culture where family members show gratitude by taking care of their parents and meeting the basic needs of older adults to feel safe, which can influence their QoL (Dorji et al., 2018; Thanakwang et al., 2014). These results are consistent with the study by You et al. (2019) who found that urban older Chinese adults have more frequent contact with their children, leading to better QoL than those living in rural areas. In fact, older adults living in a rural area have been found to experience poor access to amenities and less frequent contact with friends, which leads to lower well-being and influences QoL (Dahlberg & McKee, 2018). Conversely, Ribeiro et al. (2017) confirmed that older rural residents perceived their QoL as better than older urban residents, possibly because they were more likely to continue to work in agriculture and remain active, leading to higher self-esteem and QoL. Further, other studies indicated that the QoL of older adults did not differ between those living in urban and rural areas (Hancock & Wells, 2019; Heidari et al., 2019; Peltzer et al., 2019; Yiengprugsawan et al., 2012). Thus, evidence supporting differences between QoL in older adults living in urban and rural communities remains unclear and requires further research.

Our results showed that older adults living in urban areas had a higher perception of death and dying than those in rural areas. Only this domain differed by residential areas, we assume that the probable reason is associated with a person's perception of the aging process and place of death. The older adults who accept death as a natural stage of life may have less fear, leading to better adaptation and QoL despite potentially having multiple health conditions (Mohammadpour et al., 2018). Convenient access to healthcare services for urban residents could have an influence on their feelings of safety concerning death, whereas rural residents with remoteness from healthcare and insufficient care quality might be more worried about death and dying (Dong et al., 2019). Other domains of QoL did not differ in urban versus rural areas, which may be explained by a range of possible reasons. Evidence shows that the sociodemographic factors’ effect on QoL in older adults are differences based on the various cultures (Gobbens & Remmen, 2019). Thus, it is likely that differences were not observed because of the similar characteristics of the participants in both areas, and also due to the proximity of the urban and rural areas of Chonburi province. Older adults may experience the same effects on their QoL, which did not differ between domains. Although the death and dying domain was the only domain that differed, this nonetheless slightly influenced urban–rural differences in QoL in the current study. This finding is in line with a study that revealed that urban older Brazilian adults had a higher QoL in the facet of death and dying compared with residents in rural areas (dos Santos Tavares et al., 2014). This is a meaningful finding because living in urban areas was associated with greater access to medical care, which has been shown to improve QoL (Hsu et al., 2019). Therefore, health professionals who work with older adults, especially nurses in primary health services, should be supportive of the emotional needs of older adults. Particularly, rural residents may experience insecurity and fear about living too far from the city and not being able to access necessary care promptly (Dong et al., 2019).

In this study, HPBs had a significant positive effect on QoL of individuals in both urban and rural areas. This may be due to an effective improvement in HPBs among older adults through community activities organised by the elderly clubs that have been established across the country by the Thai government (Sutipan & Intarakamhang, 2017). This corroborates findings of other studies regarding QoL of older adults conducted in Thailand and other countries. Siboni et al. (2018) indicated that hypertensive Iranians who performed HPBs and had their blood pressure under control regardless of their living context and comorbid complications reported better QoL. HPBs include activities for controlling blood pressure and are common suggestions by professional providers focused on QoL in hypertensive older adults (Ferreira et al., 2018; Giena et al., 2018). Noticeably, empowering older adults to engage in HPBs (e.g., physical activity, healthy diet, social relationships) can influence their QoL in later life (Ferreira et al., 2018).

In urban areas, the presence of comorbidity and perceived health status were significant predictors of QoL. The results revealed that older adults with comorbidity had lower QoL scores compared with those who did not suffer from multiple chronic conditions. This is consistent with a previous study, which found that multimorbidity associated with frailty and increased disability led to a lower QoL among older adults (Rivera-Almaraz et al., 2018). Previous studies concerning urbanisation and socioeconomic disparities among older adults with hypertension found that urban residents were more likely to have unhealthy lifestyles, which increased their risk of comorbidity and led to them having a lower QoL than rural residents (Osman et al., 2019; Qin et al., 2019; Quiñones et al., 2016). With respect to the effect of perceived health status on QoL, in this study, we found that older adults with a higher perceived health status had a better QoL compared to those with poorer perceived health status. This result was supported by the study of Dumitrache et al. (2017) which indicated that older Spanish people who perceived their health as in good condition correlated with their ability to maintain health and functionality. Enhancing self-esteem to improve illness can therefore contribute to a higher life satisfaction than among those who have perceived poor health status. However, in this study, urban older adults perceived themselves as healthy, despite having more health conditions than their rural peers. This may have been caused by the accessibility of healthcare services for older people, which can improve older adults’ ability to control their blood pressure, leading to an increased QoL (Pereira et al., 2015). Similarly a previous Thai study by Seangpraw et al. (2019) found that older Thai adults who perceived their health status as moderate performed daily functions that could improve their QoL, being able to perform routine activities and participate in community activities, which promoted a good perception of health status and having a good life.

In rural areas, our findings revealed that the duration of hypertension can predict the QoL of older adults. Surprisingly, older adults with a longer duration of hypertension had higher QoL scores. Since the respondents in this study had been living with hypertension for an average of seven years, they may have had time to come to terms with the challenges of this disease. This is in line with study by Klompstra et al. (2019), who demonstrated that older adults who experienced multimorbidity had a better quality of sleep than their peers, and it affected their QoL positively over time.

### Limitations and Implications

There are some strengths and limitations in this study. To the best of our knowledge, this study may be the first study in Thailand to compare the QoL of older Thai adults with hypertension living in urban and rural areas. However, the present study also has some limitations. First, no causal relationship between various factors and QoL can be identified because of the cross-sectional design. Yet, these results provided important factors affecting QoL in older adults that healthcare providers can apply to create effective intervention targets according to their needs and cultural context. Future research should take account environmental factors (e.g., public transportation availability, leisure opportunities, medical service accessibility) related to living in urban or rural areas, as these may act as facilitators or barriers to QoL in older adults. Second, this study was conducted in one province in Thailand and, therefore, the generalizability of the results is limited. Nevertheless, this province has the highest prevalence of hypertension in Thailand and as well as rapid rates of urbanization. The setting and population of this study yield relevant results due to the effect of urbanization on older adults’ lifestyles, which increases the risk of hypertension and thus may affect QoL. Third, the demographics of this study's participants included a higher proportion of females to males as well as a higher proportion of those living with others to those living alone. These characteristics are consistent with population data from Thailand's National Statistical Office (NSO, 2020). Consequently, we considered using Welch's t-test, which assumes unequal variances, to compare QoL by gender and living arrangement in this study. Further studies need to create and examine health-promoting interventions according to the needs of older adults in distinct urban and rural areas to promote their healthy lifestyles and enhance QoL. We therefore recommend that community health care professionals focus on individualised health interventions based on sociodemographic characteristics and residential areas. Such considerations are crucial to effectively implement preventive interventions focusing on promoting health literacy, healthy habit modifications, and practicing improving QoL in older adults. We also suggested cohort investigations of different factors affecting the QoL among older adults with hypertension and a more in-depth evaluation for understanding the differences in QoL of urban and rural residents through a qualitative approach.

### Conclusion

According to our findings, the QoL of older adults with hypertension in urban districts was better than those in rural locations in Thailand. HPBs was a predictor of QoL among older adults in both areas. Understanding urban–rural gaps is important, as urban residency, presence of comorbidity, and perceived health status should be specifically addressed in individual interventions. Urban older adults may need extra support by contributing health education and supervised control of multiple chronic diseases to enhance self-care and increase their QoL. Rural residents, in turn, with shorter duration of hypertension may require more knowledge of hypertension and frequent monitoring to improve self-management of older adults. This can help older adults reduce emotional distress, improve self-confidence in taking good care of their health, and have a good QoL. Therefore, this study provides a reasonable foundation that older adults with hypertension still need more advice and health monitoring by healthcare providers. Attention should be paid based on health disparities in residential areas on QoL of older adults, which can promote their ability to live in their own home independently to maintain QoL, for as long as possible in old age.

## Notes

**Author Contributions:** List of all authors: Chonticha Chantakeeree (CC), Marjorita Sormunen (MS), Matti Estola (ME), Pornchai Jullamate (PJ), and Hannele Turunen (HT).[Table table4-00914150211050880]

**Table table4-00914150211050880:**

**Criteria**	**Author initials**
(i) Made a substantial contribution to the concept or design of the work; or acquisition, analysis or interpretation of data,	CC, MS and HT
(ii) Drafted the article or revised it critically for important intellectual content,	CC, MS, PJ, ME and HT
(iii) Approved the version to be published,	CC, MS, PJ, ME and HT

**Research Ethics and Patient Consent:** The Research Ethics Committee of the University of Eastern Finland (Statement 16/2018) approved the study, and permissions were obtained from all sub-district health promotion hospitals responsible for the elderly clubs. Written informed consent was received from all participants

**Declaration of Conflicting Interests:** The author(s) declared no potential conflicts of interest with respect to the research, authorship, and/or publication of this article.

**Funding:** The author(s) disclosed receipt of the following financial support for the research, authorship, and/or publication of this article: This work was supported by the the Human Resource Development Fund, Burapha University, Chonburi, Thailand (grant number Grant number 002/2560).

**ORCID iDs:** Chonticha Chantakeeree https://orcid.org/0000-0002-9001-1648 Hannele Turunen https://orcid.org/0000-0002-2897-9738

**Supplemental material:** Supplemental material for this article is available online.
